# Ecological and genetic impact of the 2011 Tohoku Earthquake Tsunami on intertidal mud snails

**DOI:** 10.1038/srep44375

**Published:** 2017-03-10

**Authors:** Osamu Miura, Gen Kanaya, Shizuko Nakai, Hajime Itoh, Satoshi Chiba, Wataru Makino, Tomohiro Nishimura, Shigeaki Kojima, Jotaro Urabe

**Affiliations:** 1 Faculty of Agriculture and Marine Science, Kochi University, 200 Monobe, Nankoku, Kochi 783-8502, Japan; 2 National Institute for Environmental Studies, 16-2 Onogawa, Tsukuba, Ibaraki 305-8506, Japan; 3 Department of Marine Science and Resources, College of Bioresource Sciences Nihon University, 1866 Kameino, Fujisawa, Kanagawa 252-0880, Japan; 4 Atmosphere and Ocean Research Institute, The University of Tokyo, 5-1-5, Kashiwanoha, Kashiwa, Chiba 277-8564, Japan; 5 Department of Environmental Life Sciences, Graduate School of Life Sciences, Tohoku University, Kawauchi 41, Aoba-ku, Sendai, Miyagi 980-0862, Japan; 6 Division of Ecology and Evolutionary Biology, Graduate School of Life Sciences, Tohoku University, Sendai, Miyagi 980-8578, Japan; 7 Laboratory of Aquatic Environmental Science, Faculty of Agriculture, Kochi University, Nankoku, Kochi 783-8502, Japan

## Abstract

Natural disturbances often destroy local populations and can considerably affect the genetic properties of these populations. The 2011 Tohoku Earthquake Tsunami greatly damaged local populations of various coastal organisms, including the mud snail *Batillaria attramentaria*, which was an abundant macroinvertebrate on the tidal flats in the Tohoku region. To evaluate the impact of the tsunami on the ecology and population genetic properties of these snails, we monitored the density, shell size, and microsatellite DNA variation of *B. attramentaria* for more than ten years (2005–2015) throughout the disturbance event. We found that the density of snails declined immediately after the tsunami. Bayesian inference of the genetically effective population size (*N*_e_) demonstrated that the *N*_e_ declined by 60–99% at the study sites exposed to the tsunami. However, we found that their genetic diversity was not significantly reduced after the tsunami. The maintenance of genetic diversity is essential for long-term survival of local populations, and thus, the observed genetic robustness could play a key role in the persistence of snail populations in this region which has been devastated by similar tsunamis every 500–800 years. Our findings have significant implications for understanding the sustainability of populations damaged by natural disturbances.

Large scale natural disturbances, such as fire, hurricanes, floods, drought, high wind, and large waves, often change the abundance and structure of local populations or communities^1^. Despite recognition that these disturbances play an important role in shaping biodiversity, their influence on the genetic properties of local populations is poorly appreciated^2^. A sharp and temporal decline in population size can result in the reduction of the genetically effective population size and may cause a loss of genetic diversity, a phenomenon known as a genetic bottleneck^3^,^4^,^5^,^6^. A large scale natural disturbance sometimes greatly reduces the population size, which may reduce genetic variation in local populations due to the bottleneck effect^2^.

Tsunamis associated with earthquakes are an extreme case of a natural disturbance. The Great East Japan Earthquake occurred in northeastern Japan on March 11, 2011. It reached 9.0 on the Moment Magnitude Scale, which was the largest earthquake recorded in Japan and the fourth in the world since the advent of modern seismology. Plate movement associated with the earthquake produced huge tsunamis, which hit the Pacific coastline of northeastern Japan. Tsunami heights greater than 10 m were observed along 425 km of Japan’s coast, and the maximum run-up height of the tsunami reached approximately 40 m^7^. Consequently, the tsunami greatly altered coastal landscapes in this region^8^. Many ecological studies were conducted in the areas affected by the tsunami and demonstrated that the organisms living in near-shore habitats were greatly damaged^9^,^10^. However, no studies have evaluated the impact of the tsunami on the genetic components of local populations.

Among these coastal organisms, the case of the mud snail *Batillaria attramentaria* is of particular interest. *B. attramentaria* was one of the most abundant macroinvertebrates in the intertidal mudflat of northeastern Japan, but populations of *B. attramentaria* were severely diminished and were nearly extirpated at several sites around Sendai Bay in the Tohoku region after the tsunami^11^. These severe population declines can cause a loss of genetic diversity, which potentially increases extinction risk^6^. In particular, *B. attramentaria* is a direct developer^12^, and thus the loss of genetic diversity could hardly be compensated by gene flow among local populations^13^. Since even small proportional decline in the abundance of common species can result in the disruption of ecosystem structure, function, and service^14^,^15^,^16^, ecological and genetic damages in *B. attramentaria* may lead to drastic changes in structure and function of coastal ecosystem.

To directly evaluate the ecological and genetic impacts of the tsunami, it is ideal to compare a dataset before and after the tsunami. However, since a tsunami is a rare and unpredictable event, data gathered prior to the event is only available fortuitously. We incidentally made ecological surveys on *B. attramentaria* at several intertidal mudflats in Miyagi and Fukushima Prefectures in the Tohoku region before the tsunami^11^ and preserved specimens for genetic analyses. In this study, we have expanded the ecological survey of Miura *et al*.^11^, and we further estimated population genetic indices of local populations of *B. attramentaria* for more than ten years throughout the tsunami event to evaluate the ecological and genetic impact of the tsunami on *B. attramentaria*. To our knowledge, this is the first study that simultaneously examined the ecological and genetic impact of a tsunami on marine organisms.

## Results

**Figure 1:**
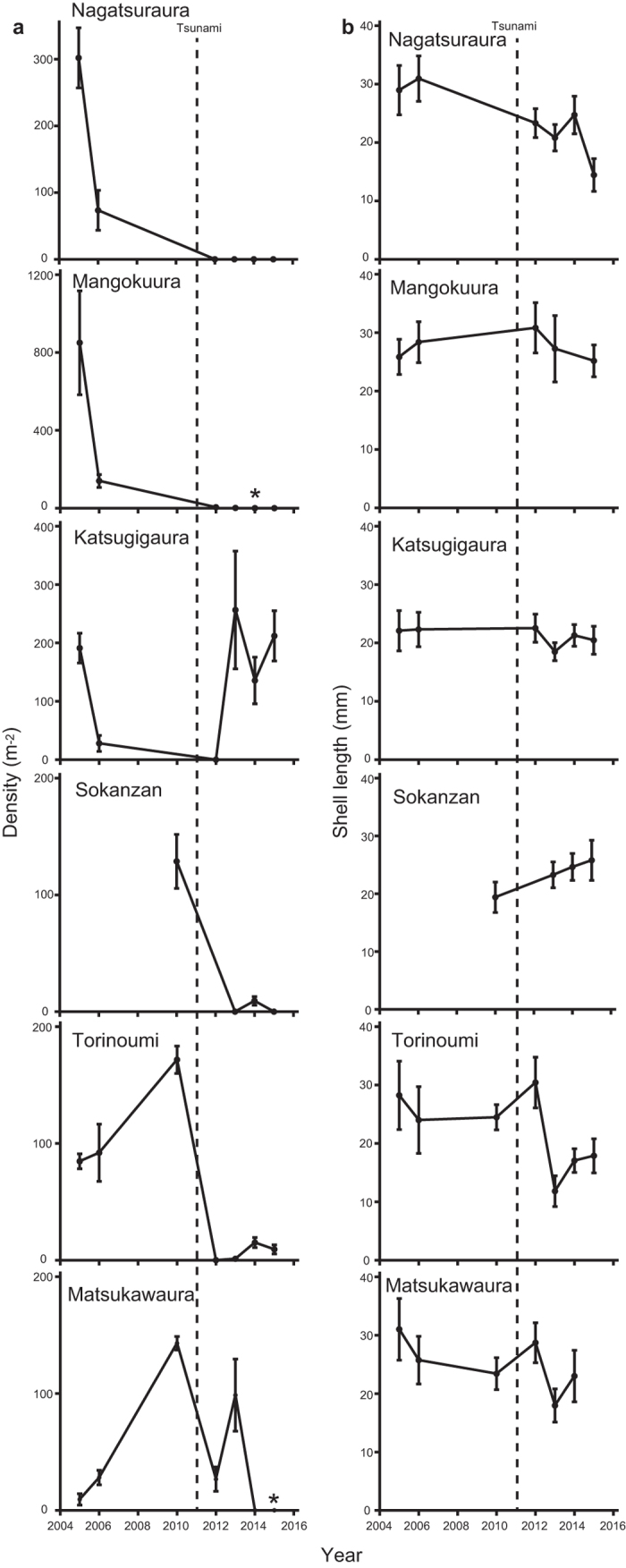
Change in ecological characters of *B. attramentaria* associated with the 2011 Tohoku Tsunami. Plots on the left are snail densities across sampling years (**a**). Asterisks indicate that no snails were found, even around the sampling sites. Error bars represent ± s.e.m. Plots on the right are shell lengths across sampling years (**b**). Error bars represent ± s.d.

*B. attramentaria* was very abundant at the six study sites before the tsunami (see Supplementary Fig. S1 for the locality), but the population was greatly reduced in 2012, one year after the tsunami (Fig. 1a). Snail densities gradually increased between 2013 and 2015, though they were not comparable to those before the tsunami at all sites, except for Katsugigaura (Fig. 1a). A significant reduction in snail density was detected after the tsunami (*F*_1,1199.3_ = 9.26, *P* = 0.02, Supplementary Table S1). The average shell size was relatively stable until 2012, but sharply decreased in 2013 (Fig. 1b) at Nagatsuraura, Katsugigaura, Torinoumi, and Matsukawaura. There were many newly recruited young snails in 2013 at these sites, which likely had been born after the tsunami. However, there were no significant reductions in shell size after the tsunami (*F*_1,104.7_ = 0.38, *P* = 0.56, Supplementary Table S1), perhaps due to variation on the effects of the tsunami among the sites and the post-tsunami growth of newly-recruited snails.

We examined microsatellite DNA data from 1600 individuals collected from six sites and seven years. All 14 loci were polymorphic (Supplementary Table S2), and there were 4–23 alleles in each locus. No loci deviated significantly from the Hardy-Weinberg equilibrium (HWE) after Bonferroni correction for multiple comparisons (Supplementary Table S3). Out of 3094 pairwise comparisons, 5 pairs showed significant linkage disequilibrium (LD) after Bonferroni correction. However, these cannot be due to physical linkage between particular microsatellite loci since no populations shared linkage disequilibrium between a pair of loci (Supplementary Table S3).

**Figure 2:**
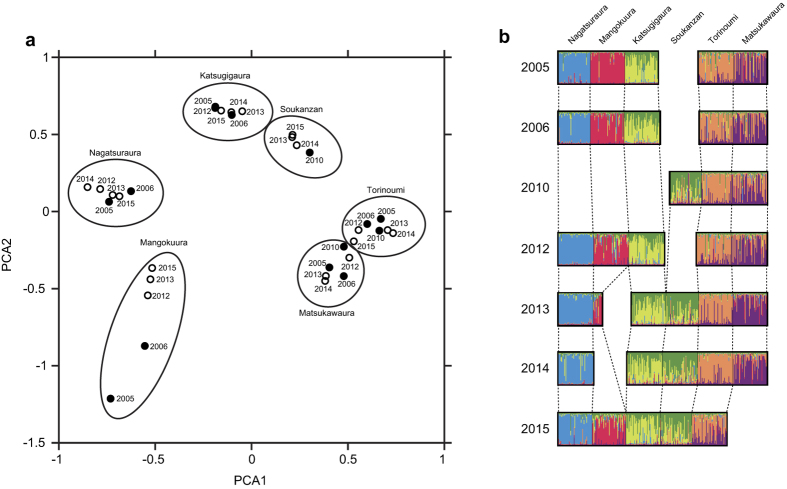
Population structure inferred from microsatellite markers. The genetic difference of each population was visualized by PCA analysis (**a**). Filled circles represent populations before the tsunami, and open circles represent populations after the tsunami. The bar plots on the right are the results of Bayesian clustering analysis for each site and each sampling year (**b**). The color of bars corresponds to cluster membership proportions.

The hierarchical analysis of molecular variance (AMOVA) estimated the percentage of overall genetic variation found among sampling years, populations within a sampling year, individuals within populations, and within individuals (Supplementary Table S4). Our results showed that while there was significant genetic structure among individuals and populations (*P* < 0.01), no significant variation was found among sampling years (*P* = 0.99). The principal component analysis (PCA) showed that the populations at each sampling site formed a cluster irrespective of sampling year (Fig. 2a). Similarly, the results of the STRUCTURE analysis indicated that the clustering pattern at each site was mostly similar across sampling years (Fig. 2b).

**Figure 3:**
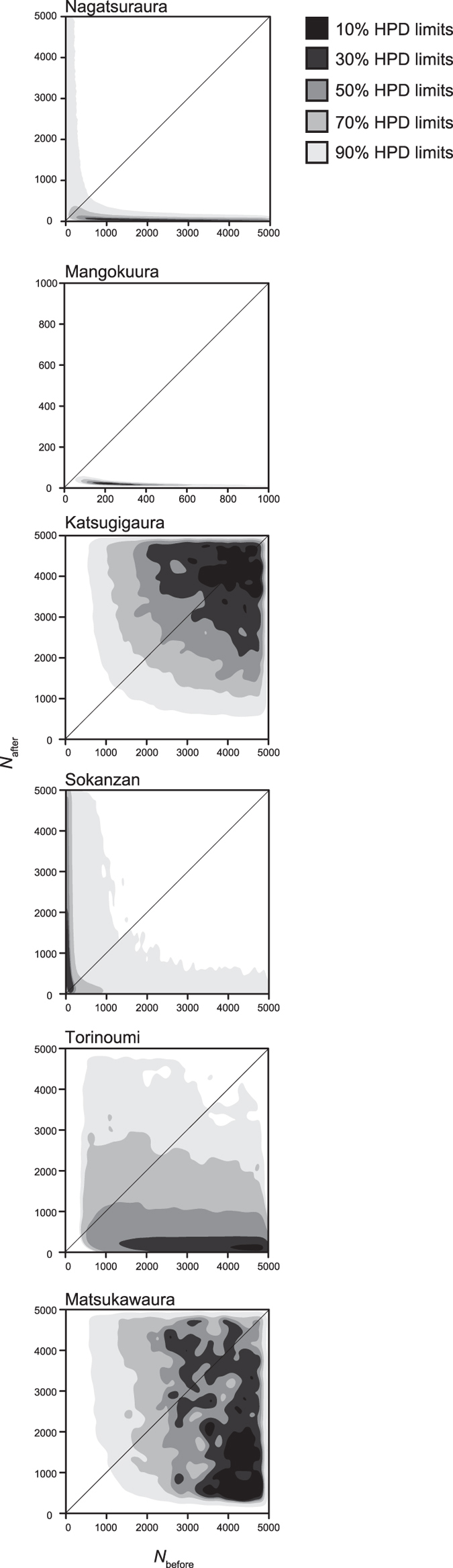
Bayesian estimation of the genetically effective population size before and after the tsunami event (*N*_before_: population size before the tsunami, *N*_after_: population size after the tsunami). The line through the middle of each box indicates *N*_before_ = *N*_after_. 10–90% highest posterior density limits (HPD) are shown in different shades of gray.

*N*_e_ estimates based on Bayesian framework^17^ exhibited positive evidence of a population bottleneck (Table 1). The posterior distribution of historical and contemporary *N*_e_ values at each site is shown in Fig. 3. The *N*_e_ at Nagatsuraura diminished by 60.3% compared to that before the tsunami (Bayes factor = 3.3), and that at Mangokuura declined by 87.7% after the earthquake (Bayes factor = 128.9). Although Bayes factors were marginally lower than the positive level (Bayes factor = 2.4), our analyses indicated that the *N*_e_ at Torinoumi declined by 98.7% after the tsunami. On the other hand, Bayesian inference suggested no clear demographic decline occurred in the populations at Katsugigaura, Sokanzan and Matsukawaura after the tsunami (Table 1).

**Figure 4:**
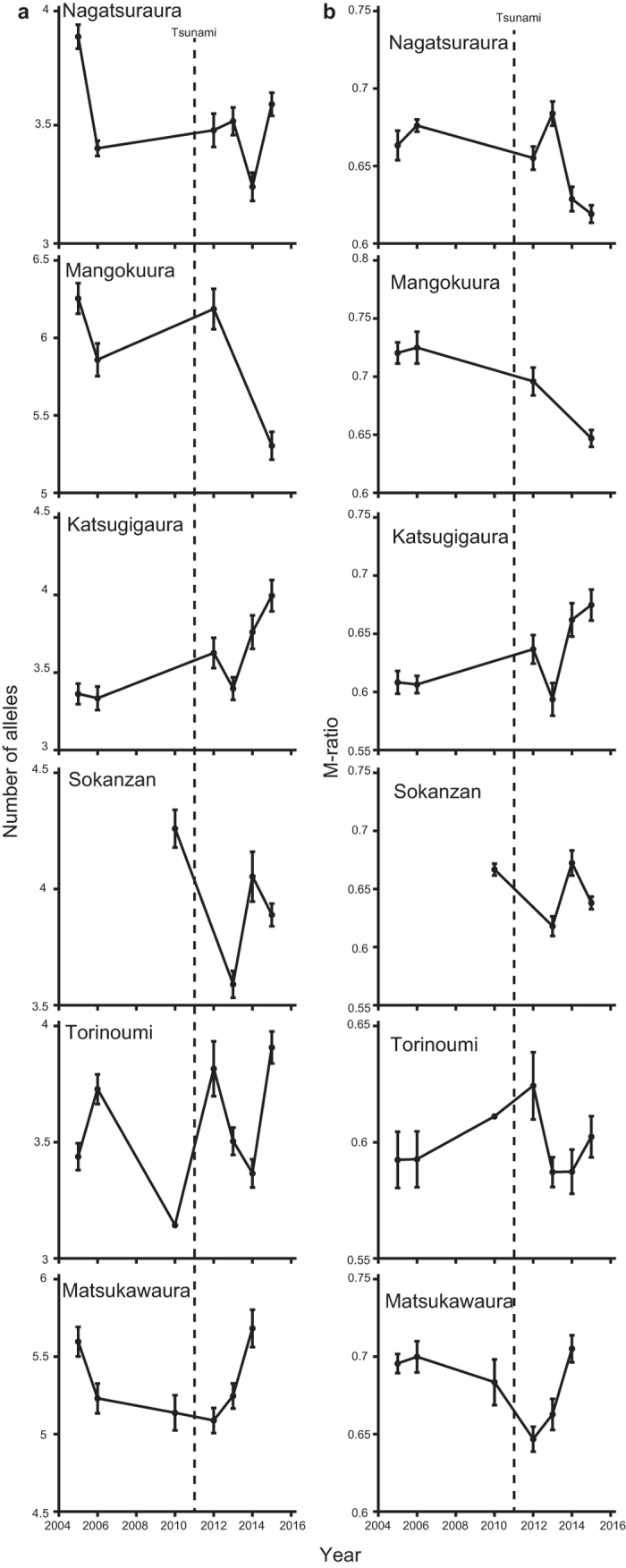
Change in the genetic diversity of *B. attramentaria* associated with the 2011 Tohoku Tsunami. Plots on the left are the rarefied allelic richness across sampling years (**a**). Plots on the right are rarefied M-ratios across sampling years (**b**). Error bars represent ± s.d.

The average expected heterozygosity and heterozygosity under mutation-drift equilibrium were relatively stable over the study period at each site (Supplementary Table S3). No significant heterozygosity excess was observed at any of the sites (Supplementary Table S3). The rarefied allelic richness did not significantly change after the tsunami at all of the study sites (Fig. 4a, Table 2). Similarly, the M-ratio did not significantly change after the tsunami at all sites (Fig. 4b, Table 2). Although we observed that allelic richness and the M-ratio fluctuated across sampling years at each site (Fig. 4a,b), these fluctuations were irregular and could be explained by pure sampling errors.

## Discussion

We demonstrated that the mud snail *B. attramentaria* was greatly declined due to the tsunami associated with the 2011 Great East Japan Earthquake. Although the estimates of temporal *N*_e_ demonstrated that several *B. attramentaria* populations in this study experienced a severe population decline, a loss of genetic diversity was not evident in any local populations at Sendai Bay. Our results exhibited that a large scale tsunami caused the mass mortality of intertidal organisms over a broad spatial scale but did not significantly affect the genetic diversity of local populations, demonstrating the robustness of genetic components against tsunami disturbances.

Our ecological monitoring showed that there was a drastic reduction in the density of *B. attramentaria* after the tsunami (Fig. 1a). On several occasions before the tsunami, the snail density exceeded 100 snails m^−2^ (Fig. 1a). The snail densities declined at several sites before the tsunami event in 2006. We consider that these declines were likely associated with seasonal behaviour of *B. attramentaria* that often move to lower shore and/or burrow under the mud during winter in Tohoku region. We therefore likely underestimated the snail density in the 2006 sampling that was exceptionally conducted in winter season (see details in Methods section). Many of the study sites were devastated by the tsunami in 2011, and snails temporally disappeared from the quadrats at Nagatsuraura, Katsugigaura, and Torinoumi (Fig. 1a). Although the population at Matsukawaura did not significantly decline immediately after the tsunami^11^, the snails at the site were suddenly crushed in 2014 (Fig. 1a) by the reclamation associated with the construction of large seawalls^8^.

Our ecological survey revealed that snail densities slightly (or greatly at Katsugigaura) increased in 2013 (Fig. 1b). We observed many newly-recruited snails in 2013, which caused a temporal decrease in the average shell length (Fig. 1b). There are two possible sources of the juvenile snails observed at these sites: (1) recruitment of juveniles from survivors at local sites (2) and colonization from surrounding populations. To determine the source of the juvenile snails observed after the tsunami, we conducted a series of population genetic analyses. The PCA and Bayesian clustering analyses consistently showed that populations before and after the tsunami were almost genetically identical (Fig. 2a,b), and the AMOVA analysis also exhibited that there was no significant variation among sampling years (Supplementary Table S4), suggesting that the young snails were produced from survivors within each site. The ecology of *B. attramentaria* explains these results, because *B. attramentaria* is a direct developer^12^ and thus has a low potential of larval dispersal^13^. Although the density of these snails was not comparable to that before the tsunami (Fig. 1a,b), our ecological and genetic surveys demonstrated that snail populations around Sendai Bay have gradually recovered from the tsunami disturbance, with breeding occurring within local populations.

The natural disturbances such as strong hurricanes are known to directly affect geographical genetic structure by mixing individuals among local populations^18^. However, our results showed that the tsunami did not affect geographical genetic structure of *B. attramentaria* (Fig. 2a,b). This result suggests that the tsunami did not facilitate the migrations of *B. attramentaria* among local populations. It is highly probable that the geographic genetic structure of many less mobile benthic organisms could remain unchanged as seen in *B. attramentaria* since they hardly return to suitable habitats after the long distance dispersal by the tsunami.

The severe population reductions and limited gene flow among the local populations may leave evidence of a bottleneck on their genetic components^5^,^19^,^20^. We first evaluated genetically effective population size (*N*_e_) which is a first genetic indicator of a reduction of actual population size and determines rates of loss of genetic diversity caused by random genetic drift^6^. We detected the declines of *N*_e_ at Nagatsuraura, Mangokuura, and Torinoumi (Table 1). The observed decline of *N*_e_ was closely associated with disturbance intensity at each site. Although snail density was nearly zero at most of the sampling sites in our 2012 sampling (Fig. 1a), there were apparent differences in the number of survivors outside the sampling quadrats. Miura *et al*.^11^ reported that snails were extremely rare at the exposed sites at Nagatsuraura and Torinoumi, even on contiguous tidal flats around the sites, whereas there was a relatively large number of survivors observed at the sheltered site such as Matsukawaura in 2012. Furthermore, abundant snails were observed at the sheltered Sokanzan site approximately four months after the tsunami^21^. Although Katsugigaura was exposed to the tsunami, there were a large number of survivors, perhaps because of the topography of the bay^11^. On the other hand, while the tsunami did not strongly affect the sampling site at Mangokuura^8^, we found that the snails at Mangokuura were greatly declined in 2013–2014 (Fig. 1a), perhaps due to subsidence^11^. These field observations indicated that severe bottlenecks occurred at Nagatsuraura, Mangokuura, and Torinoumi, compared to Sokanzan, Katsugigaura and Matsukawaura, and thus our field observations were consistent with the observed change in *N*_e_ (Table 1). Overall, concordance between these field observations and the *N*_e_ estimates strongly suggests that the tsunami and its associated events were the major factors diminishing genetically effective population size of *B. attramentaria*.

These severe declines in *N*_e_ can result in loss of genetic diversity^6^. However, the heterozygosity estimates were stable across sampling years across the tsunami event at each site, and the heterozygosity test of Luikart *et al*.^22^ demonstrated that there was no heterozygosity excess at any of the study sites (Supplementary Table S3). Allelic richness and M-ratio are more sensitive to population bottleneck in some situations^23^,^24^. However, we could not detect reductions in these indices after the tsunami (Table 2). Thus, these results demonstrated that the impact of the tsunami on genetic diversity is not quite evident, despite the apparent decline of their demography. Loss of genetic diversity was often reported in organisms that experienced severe population decline for many generations^25^,^26^,^27^,^28^. However, several other studies failed to detect a loss of genetic diversity despite severe population declines^29^,^30^. A number of factors, such as immigration, hybridization, remnant population size, and rapid recovery may weaken the strength of a genetic bottleneck^31^. In our study site, while the population size of *B. attramentaria* declined substantially after the tsunami, we were still able to find a number of snails when we searched a wider area surrounding each sampling site within the bays. This suggests that the population sizes after the tsunami might have been large enough to maintain genetic diversity in local populations. Furthermore, we observed that *B. attramentaria* has been rapidly recovering via the recruitment of a number of juveniles (Fig. 1a). This rapid recovery of population sizes could have minimized the effect of post-tsunami genetic drift.

The geological surveys conducted around the coast of the Tohoku region indicated that unusually large tsunamis, comparable to the 2011 Tohoku Tsunami, occurred in this region approximately every 500–800 years^32^, suggesting that the coastal organisms in this area have repeatedly experienced huge tsunamis in the past. Our study showed that the 2011 tsunami did not have a substantial effect on the genetics of *B. attramentaria* populations, thereby demonstrating that even a large-scale tsunami can have minimal effects on the genetic diversity of common coastal organisms. A loss of genetic diversity can have deleterious effects on population fitness and may increase the probability of extinction of local populations^6^,^33^,^34^. Therefore, the observed genetic diversity robustness may play an important role in recovery from damage caused by the tsunami and also may have contributed to persistence of snail populations after similar previous natural disturbances frequently occurred in this region.

The 2011 tsunami damaged many organisms in diverse habitats along the shore^9^. We expect that the population genetic properties of these organisms, in particular those that are more common, were also maintained after the tsunami, as we saw for *B. attramentaria*. Common species often greatly contribute biomass, energy turnover, and structure of ecosystems^14^,^15^ and thus they are essential components in coastal ecosystem. Our ecological and genetic surveys suggest that common species can relatively rapidly recover without a significant loss of genetic variation and will contribute to the sustainability of coastal ecosystem. It is highly probable that other natural disturbances with strong wave action or water flow (e.g., storm and flood) could have a similar effect on coastal ecosystem but this is a research area which needs accumulation of empirical evidence. The further compilation of a population genetic database of organisms damaged by natural disturbances will facilitate our understanding on the ecological and evolutionary effects of natural disturbances on coastal organisms.

## Methods

### Ecological surveys

Miura *et al*.^11^ reported the immediate impact of the tsunami associated with the 2011 Great East Japan Earthquake on the density and shell size of *B. attramentaria*. We expanded this ecological survey, adding a new dataset after the tsunami, in conjunction with data gathered before the tsunami (data from 2010). The dataset consists of samples collected between 2005 and 2015 at six mudflats around Sendai Bay on the Pacific coast of northeastern Japan (Nagatsuraura, Mangokuura, Katsugigaura, Sokanzan, Torinoumi, and Matsukawaura, Supplementary Fig. S1). We conducted ecological surveys during April and June in 2005, 2010, 2012, 2013, 2014, and 2015, and February 2006. We used the random quadrat method described in Miura *et al*.^11^ to estimate snail density, but a different method was used during the 2010 survey, which was independently conducted by G. K. at Sokanzan, Torinoumi, and Matsukawaura. For the 2010 survey, 3–4 quadrats (25 × 25 cm) were set on points in areas where *B. attramentaria* was present. Because the estimated density can be biased due to methodological differences, we measured snail densities using both methods at Torinoumi, and Matsukawaura, and three additional sites (Hitsugaura: 38°21′9.53″N, 141°3′34.1″E; Moune: 38°53′55.92″N, 141°37′26.28″E; Samegawa: 36°54′43.98″N, 140°49′4.31″E) in 2013 to correct potential bias. Since the density estimated by the method of G. K. was on average 4.1 times larger than that estimated by our method, we adjusted the densities measured in 2010 using this value. Snails found within quadrats were transported back to the laboratory, where their shell length (from the outer margin of the aperture to the apex of the shell) was measured, and a portion of the collected snails were preserved in 95% EtOH and/or stored at lower temperature (−30–4 °C) for molecular analyses. Often we could not find snails in any of the quadrats after the tsunami, therefore it was necessary to search a wider area surrounding each sampling site to acquire snails. However, we only searched for snails on a contiguous tidal flat, since snails inhabiting discontinuous tidal flats may be genetically distinct even within the same bay, because of their limited dispersal ability^12^,^13^. The size of a continuous tidal flat ranged from 2,500 to 60,000 m^2^. We defined a local extinction as the absence of snails within a continuous tidal flat. We used a general linear-mixed model to compare the (1) average density and (2) average shell size of *B. attramentaria* before and after the tsunami. Since we had repeatedly investigated the density and size of snails at several sites and sampling years, the sampling sites and years were treated as random effects. The interaction term between the tsunami and the sampling sites was also treated as random effect to account for the variation among the sites for susceptibility to tsunami. We ensured approximate normality of the residuals and homogeneity of variance by inspecting normal quantile plots with Lilliefors confidence limits. For the density data, the square-root transformation was used in order to satisfy the assumptions. Statistical analyses were conducted using JMP v. 9.0 (SAS Institute, Carey, NC, USA).

### Genetic analyses

Miura *et al*.^11^ estimated immediate ecological impact due to the tsunami but the genetic impact of the tsunami remains unclear. We therefore analyzed 14 microsatellite loci to evaluate genetic differences before and after the tsunami. We isolated DNA using a modified CTAB procedure^35^. We used specific primer pairs for the amplification of microsatellite loci from *B. attramentaria*^36^. For each individual, we performed two separated multiplex PCRs using the two primer sets listed in Miura *et al*.^36^ and a Type-It Microsatellite PCR Kit (Qiagen, USA). The PCR was carried out at the following parameters: activation of Hot Start DNA Taq polymerase at 95 °C for 5 min, followed by 35 cycles at 95 °C for 30 s, annealing at 56 °C for 90 s, and then elongation at 72 °C for 30 s. After the last cycle, the elongation at 60 °C was prolonged to 30 min. The PCR products were analyzed on an ABI 3100 Avant Genetic Analyzer and genotyped using GeneMapper v. 3.7 (Applied Biosystems, USA). Deviations from the HWE and LD were tested using Genepop v.4.2^37^.

We also estimated the genetic difference among populations and years by an AMOVA. Furthermore, PCA of populations was carried out to visualize the spatio-temporal genetic differences of each population. These analyses were conducted using GenoDive^38^. Finally, a Bayesian clustering algorithm implemented in the software STRUCTURE 2.3.3^39^ was employed to assign individuals to clusters using a model that allowed for admixture and correlated allele frequencies without a priori information. Since the PCA showed that the snail populations at six sites were genetically well separated, we set the number of populations to 6 for the structure analyses, and 10 iterations were analyzed with MCMC running for 1,000,000 generations at an initial burn-in of 50,000 generations. We combined values from ten different run replicates with CLUMPP^40^, and the obtained result was visualized using DISTRUCT^41^.

We further tested temporal changes in the genetically effective population size (*N*_e_) using the Bayesian method, which was incorporated in TMVP software^17^. This method is ideal for detecting changes in *N*_e_ caused by the tsunami, since it can provide the posterior distribution of *N*_e_ at the time before (*N*_e_ of the oldest sample: *N*_before_) and after the tsunami (*N*_e_ of the most recent sample: *N*_after_), based on the model of exponential growth or decline. We assumed a mean generation time of 3 years inferred from field observations of *B. attramentaria*, with a maximum effective population size of 5,000 for both ancestral and recent samples. We performed five independent runs with 1,000,000 iterations. Parameters were set at importance sample sizes of 100, thinning intervals of 10, and the size of the proposal distribution of parameter update as 0.5. We confirmed the convergence of the five runs with the Gelman–Rubin diagnostic^42^. The second half of the five runs was combined, and the posterior distributions of ancestral and recent *N*_e_ values were estimated. The results were evaluated using the Bayes factor (the proportion of MCMC iterations, where *N*_before_ > *N*_after_ divided by the proportion of iterations when *N*_before_ < *N*_after_) and by highest posterior density (HPD) limits. Bayes factors were interpreted following Jeffreys^43^, as suggested by Girod *et al*.^23^: Bayes factors greater than 10 indicate strong support, values ranging from 3 to 10 indicate substantial support, values ranging from 0.33 to 3 indicate no support, and values less than 0.33 indicate false detection. These post-processing analyses were conducted using R version 3.1.0.

Population genetic indices, such as observed heterozygosity and expected heterozygosity, were estimated for each population and each time period using GenoDive^38^. We also estimated the expected heterozygosity under mutation-drift equilibrium based on a two phase model, allowing 10% multistep mutations. A one-tailed Wilcoxon’s signed-rank test was applied to evaluate heterozygosity excess using BOTTLENECK v. 1.2.02^22^. In addition, we estimated allelic richness and the M-ratio^44^. These richness measures are sensitive to the sampling effort^45^,^46^,^47^, and we sampled a different number of individuals within populations and a different number of yearly samples before and after the tsunami. Thus, we used a resampling scheme to hierarchically rarefy measures of diversity indices to standardize the number of individuals and populations (sampled without replacement). To make the most useful comparisons, we consistently resampled 43 individuals within each population (the samples from Mangokuura in 2013 were excluded from this analysis because it contained only 12 individuals). For the overall comparison of rarefied genetic indices before and after the tsunami, resampling was conducted for 1–3 years before and after the tsunami. To generate the rarefied genetic indices, we performed 1,000 iterations using a custom-made visual basic script for Microsoft Excel and assessed the significance of the change in genetic indices using the sign test following Kalinowski^45^.

## Additional Information

**How to cite this article:** Miura, O. *et al*. Ecological and genetic impact of the 2011 Tohoku Earthquake Tsunami on intertidal mud snails. *Sci. Rep.* **7**, 44375; doi: 10.1038/srep44375 (2017).

**Publisher's note:** Springer Nature remains neutral with regard to jurisdictional claims in published maps and institutional affiliations.

## Supplementary Material

Supplementary Information

## Figures and Tables

**Table 1:** Bayesian estimation of the genetically effective population size of the sampling sites before and after the tsunami.

Site	Genetically effective population size		Rate of change (%)	Bayes factor
	Pre-tsunami	Post-tsunami		
Nagatsuraura*	75.4	29.9	−60.3	3.3
Mangokuura*	137.4	16.9	−87.7	128.9
Katsugigaura	4813.7	4821.4	0.2	1
Sokanzan	25.4	28.9	13.8	0.4
Torinoumi*	4818.1	60.7	−98.7	2.4
Matsukawaura	4819.8	4806.6	−0.3	1.2

Rates of change and Bayes factors are also estimated. The asterisks indicate the sites severely affected by the tsunami or subsidence.

**Table 2:** Rarefied allelic richness and M-ratios estimated before and after the tsunami event.

Site	Number of samples used in hierarchical rarefaction analysis		Allelic richness ± SD		M-ratio ± SD	
	Years per block	Samples in total	Before	After	Before	After
Nagatsuraura*	2	172	4.24 ± 0.05	3.90 ± 0.08	0.70 ± 0.01	0.66 ± 0.02
Mangokuura*	2	172	6.96 ± 0.10	6.80 ± 0.11	0.76 ± 0.01	0.72 ± 0.01
Katsugigaura	2	172	3.78 ± 0.07	4.48 ± 0.22	0.65 ± 0.01	0.70 ± 0.02
Sokanzan	1	86	4.26 ± 0.08	3.84 ± 0.21	0.67 ± 0.01	0.64 ± 0.02
Torinoumi*	3	258	4.08 ± 0.07	4.44 ± 0.15	0.63 ± 0.01	0.65 ± 0.01
Matsukawaura	3	258	6.52 ± 0.10	6.92 ± 0.10	0.75 ± 0.01	0.74 ± 0.01

The number of samples (populations/individuals) used in hierarchical resampling models is also shown. The asterisks indicate the sites severely affected by the tsunami or subsidence. The rarefied allelic richness and M-ratio did not significantly change after the tsunami at all of the study sites (*P* > 0.05). Note that the estimated M-ratio values can be inflated because of the presence of an imperfect repeat in some microsatellite loci^36^.
