# Supplementary material for: Ecological and genetic impact of the 2011 Tohoku Earthquake Tsunami on intertidal mud snails
Source: Sci Rep. 2017 Mar 10;7:44375. doi: 10.1038/srep44375 (PMC5345064; doi:10.1038/srep44375)
Supplement: Supplementary Information [file srep44375-s1.pdf]

Supplementary Information for:

Ecological and genetic impact of the 2011 Tohoku Earthquake Tsunami on intertidal mud snails

Osamu Miura, Gen Kanaya, Shizuko Nakai, Hajime Itoh, Satoshi Chiba, Wataru Makino, Tomohiro Nishimura, Shigeaki Kojima and Jotaro Urabe

This supplement contains:

Supplementary Figure S1

Supplementary Table S1

Supplementary Table S2

Supplementary Table S3

Supplementary Table S4

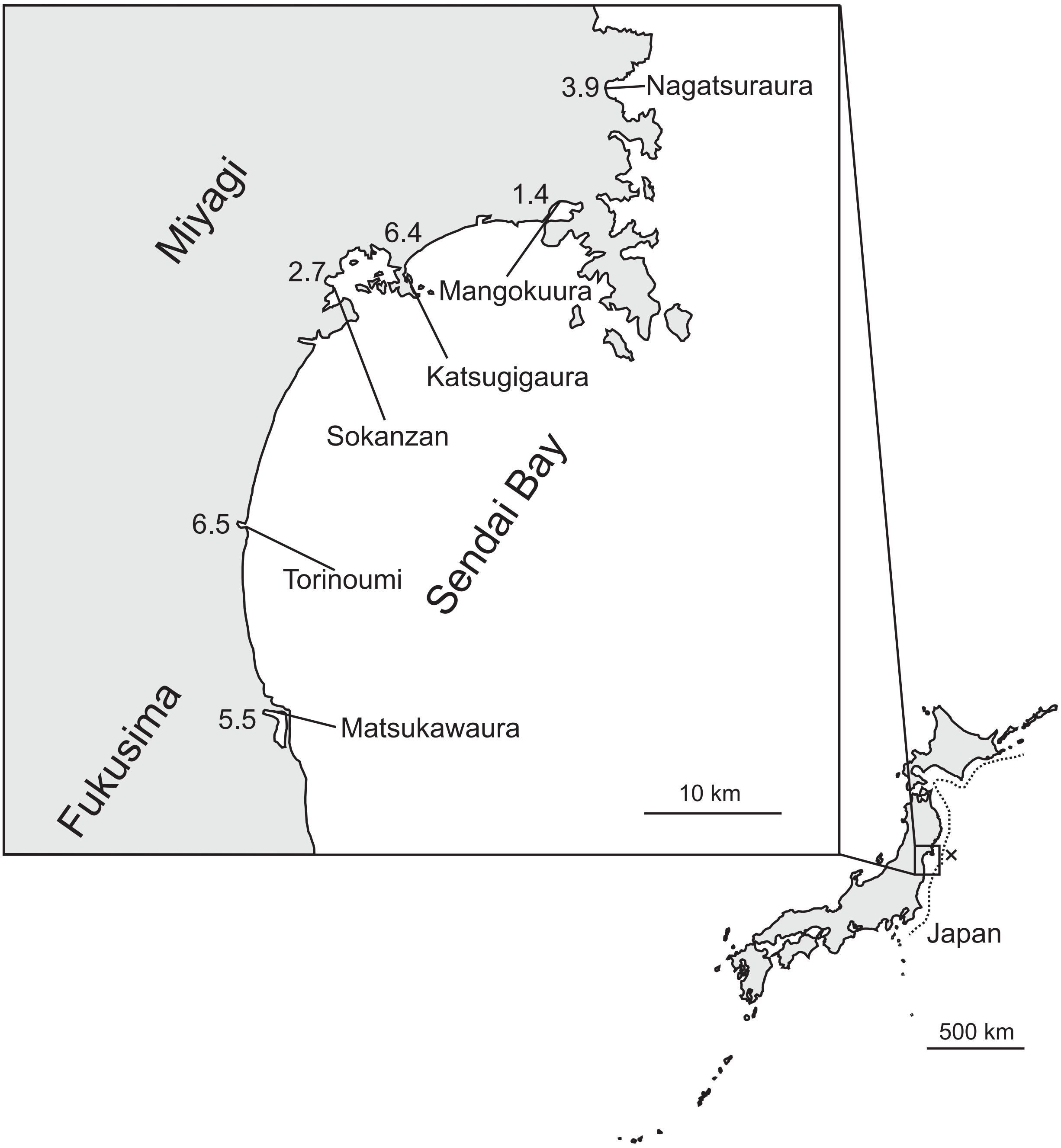

Supplementary Figure S1. Sampling sites in Tohoku region (Nagatsuraura site: 38°33′26.7″N, 141°27′27.7″E; Mangokuura site: 38°25′39.6″N, 141°22′44.3″E; Katsugigaura site: 38°20′52.9″N, 141°09′16.0″E; Sokanzan site: 38°21'8.5"N, 141° 3'34.1"E; Torinoumi site: 38°01′51.1″N, 140°54′31.4″E; Matsukawaura site: 37°49′22.9″N, 140°57′23.2″E). Numbers beside sampling sites indicate inundation height of tsunami (m)(recorded at closest location to each site), based on Mori et al. (2011). x: epicenter of the 2011 Tohoku Earthquake; dotted line: region hit by the large tsunami. The maps were created by Haku-chizu KenMap v.9.11 (http://www5b.biglobe.ne.jp/~t-kamada/CBuilder/kenmap.htm, freeware approved by Geospatial Information Authority of Japan with approval No. 149 in 2002) and modified in Adobe Illustrator CS5.

Supplementary Table S1. Summary of the general liner mixed model analyses on the effect of the tsunami on the snail density (a) and the shell size (b). The site, year, and tsunami x site are treated as random effects and tsunami is treated as a fixed effect.

(a) Snail density

Source	SS	MS (num)	DF (num)	F-value	P-value
Tsunami	1199.27	1199.27	1	9.26	0.02
Site (random)	3280.19	656.04	5	0.81	0.59
Year (random)	2450.01	490.00	5	9.26	< 0.0001
Tsunami x Site (random)	4026.65	805.33	5	30.24	< 0.0001

(b) Shell size

Source	SS	MS (num)	DF (num)	F-value	P-value
Tsunami	104.68	104.68	1	0.38	0.56
Site (random)	19243.30	3848.66	5	1.36	0.37
Year (random)	21492.50	4298.49	5	246.75	< 0.0001
Tsunami x Site (random)	14172.20	2834.44	5	162.71	< 0.0001

Supplementary Table S2. Genetic variation of each 14 microsatellite markers in each population. Number of the samples analyzed (N), the number of alleles (N_A), observed heterozygosity (H_O), expected heterozygosity (H_E) were estimated for each loci.

Sampling year	Site		BA0743	BA2553	BM5001	BA7669	BM0558	BA1521	BA5409	BM5507	BA8329	BM0786	BA2364	BA8415	BA0807	BA8253
	Nagatsuraura	N	45													
		N_A	3	6	4	3	2	8	5	6	3	2	3	4	2	4
		H_O	0.04	0.44	0.64	0.31	0.02	0.64	0.64	0.69	0.64	0.53	0.22	0.51	0.38	0.62
		H_E	0.04	0.44	0.65	0.38	0.02	0.62	0.65	0.62	0.60	0.50	0.24	0.49	0.31	0.68
	Mangokuura	N	49													
		N_A	4	8	3	4	6	19	8	3	6	3	5	12	4	5
		H_O	0.22	0.51	0.51	0.53	0.65	0.90	0.80	0.29	0.13	0.51	0.55	0.84	0.76	0.38
		H_E	0.21	0.47	0.62	0.57	0.61	0.89	0.72	0.27	0.17	0.49	0.55	0.84	0.67	0.55
	Katsugigaura	N	47													
		N_A	3	2	4	4	3	6	4	4	3	3	2	5	2	3
2005		H_O	0.34	0.09	0.62	0.68	0.13	0.60	0.43	0.57	0.30	0.36	0.34	0.21	0.28	0.47
		H_E	0.37	0.08	0.65	0.54	0.12	0.59	0.43	0.58	0.29	0.34	0.34	0.28	0.27	0.47
	Torinoumi	N	49													
		N_A	2	3	3	3	4	6	3	4	2	3	5	4	3	4
		H_O	0.12	0.06	0.55	0.49	0.47	0.78	0.67	0.59	0.06	0.18	0.25	0.18	0.39	0.67
		H_E	0.22	0.06	0.54	0.55	0.44	0.77	0.54	0.64	0.06	0.17	0.25	0.21	0.45	0.68
	Matsukawaura	N	47													
		N_A	2	5	3	4	7	11	11	5	3	3	7	12	3	4
		H_O	0.13	0.19	0.60	0.51	0.66	0.87	0.61	0.75	0.36	0.32	0.45	0.47	0.34	0.57
		H_E	0.12	0.20	0.51	0.63	0.60	0.84	0.66	0.76	0.40	0.28	0.53	0.53	0.39	0.61
	Nagatsuraura	N	45													
		N_A	3	5	3	4	1	4	4	6	3	2	2	5	2	4
		H_O	0.13	0.33	0.67	0.47	0.00	0.53	0.67	0.53	0.69	0.53	0.16	0.58	0.27	0.67
		H_E	0.13	0.32	0.66	0.44	0.00	0.52	0.67	0.57	0.61	0.48	0.15	0.56	0.30	0.70
	Mangokuura	N	48													
		N_A	5	7	5	4	7	16	6	4	4	3	4	12	4	3
		H_O	0.25	0.38	0.71	0.52	0.58	0.83	0.75	0.48	0.08	0.50	0.44	0.77	0.69	0.46
		H_E	0.26	0.37	0.66	0.56	0.61	0.88	0.71	0.59	0.20	0.50	0.56	0.81	0.62	0.53
	Katsugigaura	N	50													
		N_A	4	3	4	4	2	6	3	4	2	2	3	6	2	3
2006		H_O	0.48	0.14	0.70	0.38	0.08	0.62	0.54	0.56	0.32	0.36	0.38	0.20	0.30	0.64
		H_E	0.43	0.18	0.65	0.52	0.08	0.65	0.47	0.56	0.30	0.32	0.34	0.24	0.29	0.56
	Torinoumi	N	47													
		N_A	2	2	3	3	5	6	5	5	2	3	5	5	3	4
		H_O	0.11	0.02	0.55	0.57	0.57	0.83	0.70	0.60	0.06	0.19	0.30	0.26	0.53	0.79
		H_E	0.10	0.02	0.51	0.55	0.50	0.77	0.62	0.68	0.06	0.18	0.29	0.29	0.54	0.69
	Matsukawaura	N	48													
		N_A	3	6	4	4	6	9	8	6	3	3	6	10	3	4
		H_O	0.13	0.15	0.56	0.50	0.56	0.81	0.52	0.71	0.13	0.27	0.46	0.50	0.56	0.46
		H_E	0.12	0.16	0.53	0.63	0.54	0.82	0.61	0.74	0.16	0.33	0.53	0.45	0.54	0.61
	Sokanzan	N	44													
		N_A	4	4	5	5	4	7	4	5	3	3	3	6	3	4
		H_O	0.32	0.18	0.77	0.40	0.30	0.73	0.54	0.71	0.11	0.05	0.30	0.23	0.25	0.71
		H_E	0.32	0.21	0.69	0.47	0.30	0.73	0.53	0.68	0.15	0.09	0.29	0.23	0.32	0.61
	Torinoumi	N	43													
		N_A	2	1	2	3	3	7	4	4	2	2	4	3	3	4
2010		H_O	0.23	0.00	0.44	0.58	0.51	0.79	0.56	0.67	0.02	0.16	0.33	0.28	0.47	0.67
		H_E	0.28	0.00	0.47	0.58	0.47	0.77	0.53	0.68	0.02	0.15	0.29	0.25	0.51	0.69
	Matsukawaura	N	50													
		N_A	4	6	3	4	7	10	8	5	3	3	4	10	4	4
		H_O	0.24	0.18	0.42	0.62	0.48	0.84	0.52	0.64	0.16	0.22	0.44	0.40	0.40	0.40
		H_E	0.22	0.17	0.48	0.60	0.53	0.79	0.58	0.74	0.17	0.23	0.50	0.42	0.44	0.56
	Nagatsuraura	N	50													
		N_A	3	5	3	3	1	7	4	5	3	2	2	6	2	4
		H_O	0.16	0.40	0.58	0.36	0.00	0.42	0.48	0.42	0.70	0.52	0.32	0.56	0.30	0.62
		H_E	0.15	0.42	0.66	0.39	0.00	0.45	0.56	0.56	0.64	0.48	0.27	0.52	0.34	0.66
	Mangokuura	N	50													
		N_A	3	7	4	4	7	19	7	5	5	2	5	13	4	5
		H_O	0.34	0.52	0.64	0.38	0.50	0.90	0.68	0.64	0.19	0.26	0.48	0.62	0.52	0.53
		H_E	0.31	0.49	0.65	0.43	0.52	0.88	0.77	0.67	0.28	0.42	0.54	0.76	0.59	0.59
	Katsugigaura	N	50													
		N_A	3	3	4	3	7	5	4	4	4	2	3	5	2	4
2012		H_O	0.32	0.16	0.60	0.48	0.16	0.68	0.42	0.52	0.32	0.38	0.34	0.14	0.38	0.52
		H_E	0.30	0.15	0.67	0.50	0.15	0.62	0.50	0.54	0.31	0.38	0.37	0.17	0.34	0.55
	Torinoumi	N	49													
		N_A	2	2	3	4	3	6	6	6	3	3	4	6	3	4
		H_O	0.14	0.02	0.63	0.61	0.41	0.82	0.51	0.78	0.04	0.27	0.29	0.37	0.45	0.45
		H_E	0.23	0.02	0.56	0.58	0.40	0.78	0.62	0.69	0.04	0.24	0.27	0.38	0.44	0.65
	Matsukawaura	N	50													
		N_A	3	5	3	5	6	10	8	5	4	2	5	9	4	4
		H_O	0.32	0.14	0.48	0.64	0.52	0.78	0.58	0.64	0.26	0.34	0.42	0.56	0.36	0.50
		H_E	0.34	0.17	0.55	0.66	0.48	0.80	0.56	0.73	0.25	0.34	0.42	0.54	0.52	0.64

Continued.

Sampling year	Site		BA0743	BA2553	BM5001	BA7669	BM0558	BA1521	BA5409	BM5507	BA8329	BM0786	BA2364	BA8415	BA0807	BA8253
	Nagatsuraura	N	49													
		N_A	3	5	3	3	1	6	4	6	4	2	2	5	2	4
		H_O	0.22	0.49	0.67	0.41	0.00	0.45	0.59	0.61	0.63	0.43	0.16	0.61	0.29	0.63
		H_E	0.21	0.49	0.64	0.35	0.00	0.51	0.60	0.56	0.65	0.46	0.15	0.51	0.33	0.69
	Mangokuura	N	12													
		N_A	3	4	3	4	4	8	6	4	2	2	3	5	3	4
		H_O	0.17	0.58	0.42	0.42	0.42	0.73	0.83	0.42	0.25	0.42	0.33	0.67	0.42	0.58
		H_E	0.42	0.48	0.48	0.58	0.37	0.84	0.80	0.64	0.35	0.43	0.53	0.67	0.55	0.66
	Katsugigaura	N	49													
		N_A	2	3	5	4	5	5	3	4	3	3	2	5	2	3
		H_O	0.31	0.18	0.71	0.61	0.12	0.63	0.57	0.61	0.27	0.25	0.27	0.20	0.25	0.53
		H_E	0.39	0.17	0.68	0.53	0.12	0.63	0.57	0.55	0.29	0.25	0.26	0.21	0.30	0.52
2013	Sokanzan	N	46													
		N_A	3	2	4	3	3	9	5	4	3	2	3	5	2	3
		H_O	0.39	0.09	0.70	0.52	0.22	0.80	0.50	0.63	0.13	0.09	0.26	0.22	0.20	0.63
		H_E	0.42	0.08	0.65	0.49	0.23	0.72	0.54	0.65	0.12	0.08	0.23	0.22	0.21	0.57
	Torinoumi	N	49													
		N_A	2	2	3	3	5	7	3	4	4	2	4	4	3	4
		H_O	0.14	0.04	0.47	0.53	0.49	0.71	0.55	0.71	0.12	0.14	0.33	0.51	0.45	0.61
		H_E	0.13	0.04	0.46	0.57	0.47	0.75	0.53	0.66	0.12	0.13	0.29	0.49	0.40	0.65
	Matsukawaura	N	48													
		N_A	2	6	3	4	7	10	8	6	3	3	6	10	3	5
		H_O	0.27	0.23	0.48	0.60	0.54	0.88	0.52	0.67	0.48	0.27	0.50	0.40	0.35	0.46
		H_E	0.24	0.23	0.53	0.65	0.57	0.83	0.69	0.73	0.48	0.30	0.52	0.48	0.56	0.54
	Nagatsuraura	N	49													
		N_A	3	5	3	3	2	3	5	5	3	2	3	3	2	4
		H_O	0.27	0.49	0.55	0.49	0.12	0.35	0.51	0.57	0.47	0.59	0.43	0.59	0.37	0.86
		H_E	0.24	0.47	0.61	0.40	0.12	0.36	0.66	0.62	0.48	0.50	0.43	0.54	0.41	0.67
	Katsugigaura	N	50													
		N_A	2	5	5	3	5	6	4	4	3	3	3	5	4	3
		H_O	0.22	0.16	0.68	0.54	0.10	0.68	0.52	0.56	0.30	0.28	0.20	0.18	0.26	0.56
		H_E	0.29	0.21	0.68	0.55	0.10	0.63	0.50	0.57	0.26	0.32	0.25	0.19	0.35	0.52
	Sokanzan	N	50													
		N_A	4	2	3	4	6	8	6	6	3	2	4	4	3	4
2014		H_O	0.30	0.08	0.66	0.46	0.28	0.68	0.48	0.66	0.20	0.06	0.20	0.30	0.22	0.44
		H_E	0.35	0.08	0.65	0.51	0.30	0.71	0.55	0.65	0.22	0.06	0.22	0.27	0.39	0.56
	Torinoumi	N	50													
		N_A	2	2	2	3	3	5	6	4	2	2	4	6	3	4
		H_O	0.28	0.08	0.40	0.58	0.46	0.80	0.48	0.66	0.06	0.12	0.20	0.44	0.34	0.72
		H_E	0.24	0.08	0.49	0.57	0.49	0.73	0.54	0.62	0.06	0.11	0.20	0.40	0.41	0.69
	Matsukawaura	N	48													
		N_A	2	7	3	4	6	13	9	7	4	3	6	10	3	5
		H_O	0.15	0.17	0.65	0.58	0.38	0.73	0.53	0.67	0.13	0.44	0.52	0.65	0.48	0.63
		H_E	0.14	0.18	0.54	0.61	0.50	0.83	0.67	0.75	0.16	0.37	0.51	0.61	0.58	0.61
	Nagatsuraura	N	48													
		N_A	3	3	3	3	2	6	4	7	3	2	2	7	2	4
		H_O	0.19	0.29	0.67	0.40	0.04	0.58	0.54	0.63	0.54	0.38	0.17	0.56	0.46	0.81
		H_E	0.18	0.37	0.64	0.47	0.04	0.55	0.68	0.64	0.65	0.46	0.15	0.54	0.42	0.71
	Mangokuura	N	48													
		N_A	3	6	4	5	4	15	7	5	4	2	4	9	4	4
		H_O	0.27	0.25	0.71	0.48	0.17	0.65	0.54	0.63	0.19	0.58	0.38	0.58	0.65	0.33
		H_E	0.27	0.29	0.68	0.49	0.24	0.81	0.69	0.71	0.28	0.46	0.50	0.69	0.64	0.55
	Katsugigaura	N	49													
		N_A	3	2	6	3	6	6	4	4	4	2	4	6	4	4
2015		H_O	0.37	0.10	0.74	0.47	0.12	0.69	0.35	0.59	0.20	0.37	0.29	0.25	0.35	0.57
		H_E	0.39	0.10	0.67	0.53	0.12	0.65	0.47	0.54	0.27	0.30	0.30	0.28	0.38	0.54
	Sokanzan	N	45													
		N_A	3	2	4	5	5	6	4	6	2	2	3	6	3	4
		H_O	0.27	0.13	0.62	0.56	0.09	0.62	0.27	0.60	0.16	0.07	0.20	0.22	0.33	0.58
		H_E	0.34	0.16	0.64	0.56	0.15	0.63	0.57	0.69	0.15	0.07	0.19	0.28	0.28	0.56
	Torinoumi	N	49													
		N_A	2	3	3	3	5	7	5	5	3	3	4	6	3	4
		H_O	0.12	0.08	0.45	0.69	0.14	0.80	0.53	0.63	0.08	0.27	0.27	0.41	0.45	0.74
		H_E	0.12	0.10	0.53	0.60	0.23	0.79	0.56	0.66	0.08	0.27	0.29	0.37	0.51	0.62

Supplementary Table S3. Average heterozygosity across 14 microsatellite markers in each population. Number of the samples analyzed (N), observed heterozygosity (H_O), expected heterozygosity (H_E), heterozygosity at expected under mutation-drift equilibrium (H_{EQ}), the number of loci significantly deviated from Hardy-Weinberg equilibrium (HWE), and pairs of loci exhibiting significant linkage disequilibrium (LD) were estimated for each population. No populations exhibited significant heterozygosity excess ($H_E > H_{EQ}$) and no loci exhibited significant deviation from HWE.

Year	Site	N	N_A	H_O	H_E	H_{EQ}	HWE	LD
2005	Nagatsuraura	45	3.93	0.45	0.45	0.49	0	-
	Mangokuura	49	6.43	0.54	0.55	0.64	0	BA5409-BM5507
	Katsugigaura	47	3.43	0.39	0.38	0.45	0	-
	Torinoumi	49	3.50	0.39	0.40	0.47	0	-
	Matsukawaura	47	5.71	0.49	0.50	0.59	0	-
2006	Nagatsuraura	45	3.43	0.44	0.44	0.44	0	-
	Mangokuura	48	6.00	0.53	0.56	0.63	0	-
	Katsugigaura	50	3.43	0.41	0.40	0.44	0	-
	Torinoumi	47	3.79	0.43	0.41	0.49	0	-
	Matsukawaura	48	5.36	0.45	0.48	0.61	0	-
2010	Sokanzan	44	4.29	0.40	0.40	0.56	0	-
	Torinoumi	43	3.14	0.41	0.41	0.45	0	-
	Matsukawaura	50	5.36	0.43	0.46	0.61	0	-
2012	Nagatsuraura	50	3.57	0.42	0.44	0.44	0	-
	Mangokuura	50	6.43	0.51	0.56	0.63	0	BM0558-BA8415, BA5409-BA8415
	Katsugigaura	50	3.79	0.39	0.40	0.49	0	-
	Torinoumi	49	3.93	0.41	0.42	0.50	0	-
	Matsukawaura	50	5.21	0.47	0.50	0.60	0	-
2013	Nagatsuraura	49	3.57	0.44	0.44	0.45	0	-
	Mangokuura	12	3.93	0.48	0.56	0.58	0	-
	Katsugigaura	49	3.50	0.39	0.39	0.46	0	-
	Sokanzan	46	3.64	0.38	0.37	0.46	0	-
	Torinoumi	49	3.57	0.42	0.41	0.46	0	-
	Matsukawaura	48	5.43	0.48	0.53	0.60	0	-
2014	Nagatsuraura	49	3.29	0.48	0.46	0.44	0	BA0743-BA8415, BM5507-BA8253
	Katsugigaura	50	3.93	0.37	0.39	0.52	0	-
	Sokanzan	50	4.21	0.36	0.39	0.52	0	-
	Torinoumi	50	3.43	0.40	0.40	0.43	0	-
	Matsukawaura	48	5.86	0.48	0.50	0.61	0	-
2015	Nagatsuraura	48	3.64	0.45	0.46	0.45	0	-
	Mangokuura	48	5.43	0.46	0.52	0.60	0	-
	Katsugigaura	49	4.14	0.39	0.40	0.53	0	-
	Sokanzan	45	3.93	0.34	0.38	0.50	0	-
	Torinoumi	49	4.00	0.40	0.41	0.52	0	-

Supplementary Table S4. Analysis of molecular variance (AMOVA) of *B. attramentaria* among geographic locations and collections between 2005 and 2015.

Source of variation	df	%var	Fixation index	P-value
Within individuals	1600	0.877	0.123	
Among individuals	1566	0.027	0.029	0.001
Among populations	27	0.113	0.111	0.001
Among years	6	-0.016	-0.016	0.999
